# Publisher Correction: Ticks parasitised feathered dinosaurs as revealed by Cretaceous amber assemblages

**DOI:** 10.1038/s41467-018-02913-w

**Published:** 2018-01-30

**Authors:** Enrique Peñalver, Antonio Arillo, Xavier Delclòs, David Peris, David A. Grimaldi, Scott R. Anderson, Paul C. Nascimbene, Ricardo Pérez-de la Fuente

**Affiliations:** 1 Museo Geominero, Instituto Geológico y Minero de España, 28003 Madrid, Spain; 2 Departamento de Zoología y Antropología Física, Facultad de Biología, Universidad Complutense, 28040 Madrid, Spain; 3 Departament de Dinàmica de la Terra i de l’Oceà and Institut de Recerca de la Biodiversitat (IRBio), Facultat de Ciències de la Terra, Universitat de Barcelona, 08028 Barcelona, Spain; 4 Departament de Ciències Agràries i del Medi Natural, Universitat Jaume I, 12071 Castelló de la Plana, Spain; 5 Division of Invertebrate Zoology, American Museum of Natural History, New York, NY 10021 USA; 6 Independent Researcher, Moon Township, USA; 7 Oxford University Museum of Natural History, Parks Road, Oxford, OX1 3PW UK

Correction to: *Nature Communications* 10.1038/s41467-017-01550-z, Article published online 12 December 2017

The originally published version of this Article was updated shortly after publication to add the word ‘Ticks’ to the title, following its inadvertent removal during the production process. This has now been corrected in both the PDF and HTML versions of the Article.

