# Supplementary material for: Ticks parasitised feathered dinosaurs as revealed by Cretaceous amber assemblages
Source: Nat Commun. 2017 Dec 12;8:1924. doi: 10.1038/s41467-017-01550-z (PMC5727220; doi:10.1038/s41467-017-01550-z)
Supplement: Supplementary file 1 — Supplementary Information [file 41467_2017_1550_MOESM1_ESM.pdf]

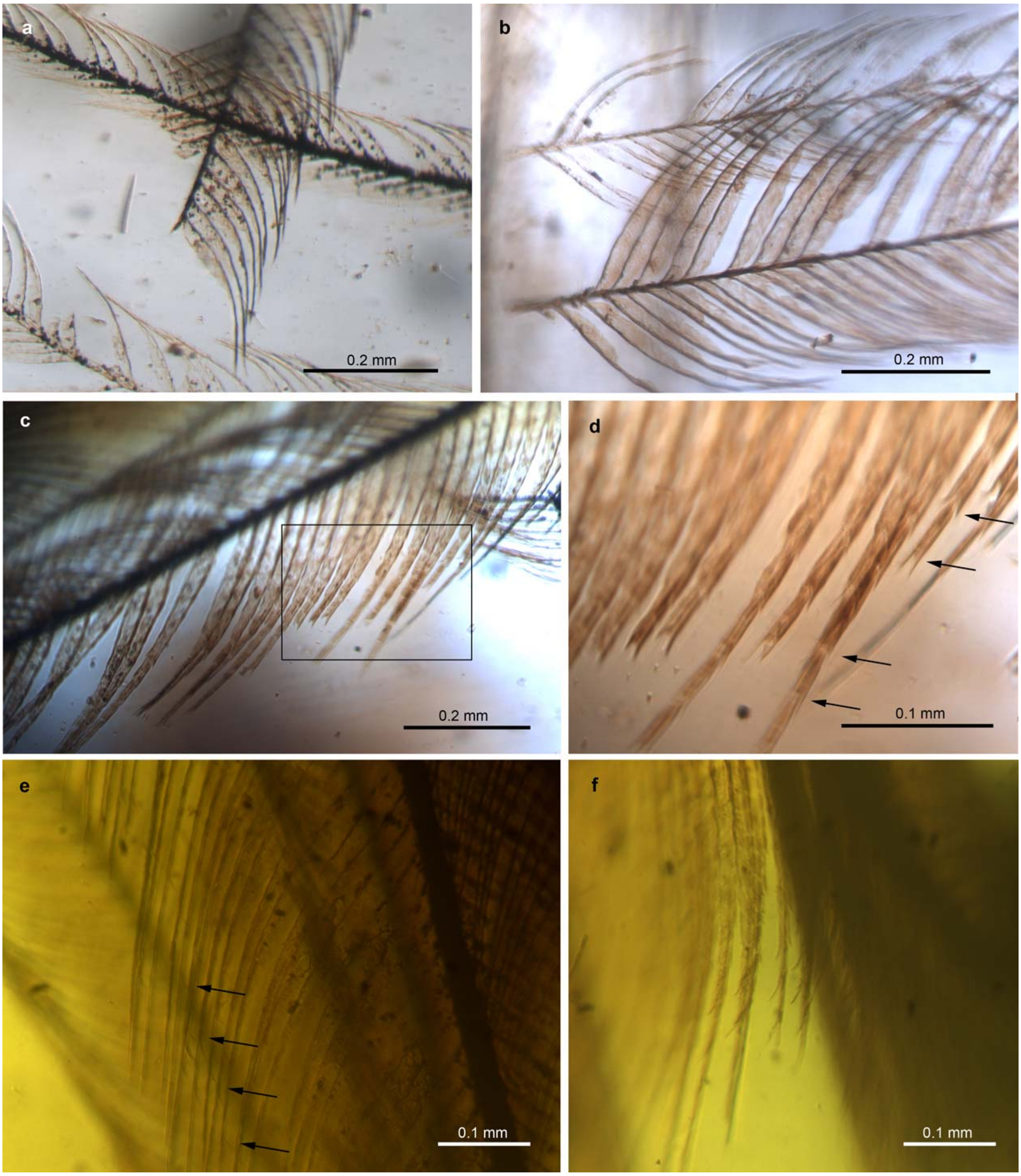

Supplementary Figure 1. Feather remains in the ixodid-bearing amber piece (Bu JZC-F18). **a** Three barbs of the semicomplete pennaceous feather showing damage. **b** Two isolated barbs from a different feather. **c** Barb of the semicomplete pennaceous feather, showing barbules located distally along the barb. **d** Detail of the barbules and their spined nodes (arrows) and internodes (inset in **c**). **e** Another barb of the semicomplete pennaceous feather, showing barbules located medially along the barb with pennula showing hooklets on one of their sides (arrows). **f** Hooklets in a different set of pennula from a different barb from the semicomplete pennaceous feather.

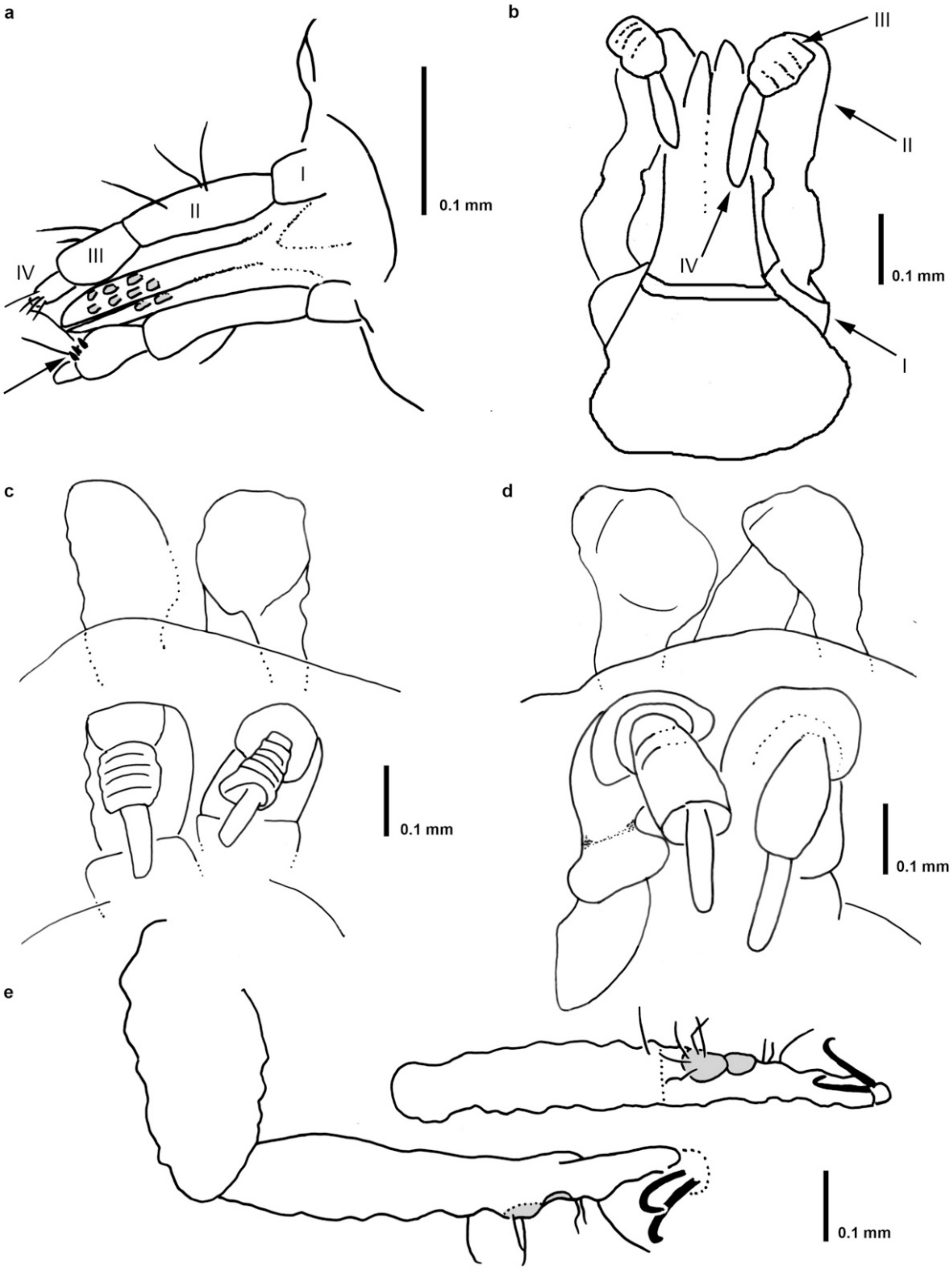

Supplementary Figure 2. Camera lucida drawings showing some anatomical features of the ixodid and deinocrotonid specimens. **a** Capitulum in ventral view of the ixodid *Cornupalpatum burmanicum* (AMNH Bu JZC-F18); hypostome teeth in grey, arrow indicates the ventrolateral pretarsal claws in the third palpal segment. **b** Capitulum in ventral view of the deinocrotonid paratype male (AMNH Bu-SA5b). **c**–**d** Palpi in dorsal (top) and ventral (bottom) views in the deinocrotonid holotype (AMNH Bu-SA5a) and the allotype (CM 63,007), respectively. **e** Two views of the Haller’s organ (in grey) in the deinocrotonid holotype right tarsus I. Panels **b**–**e** to the same scale.

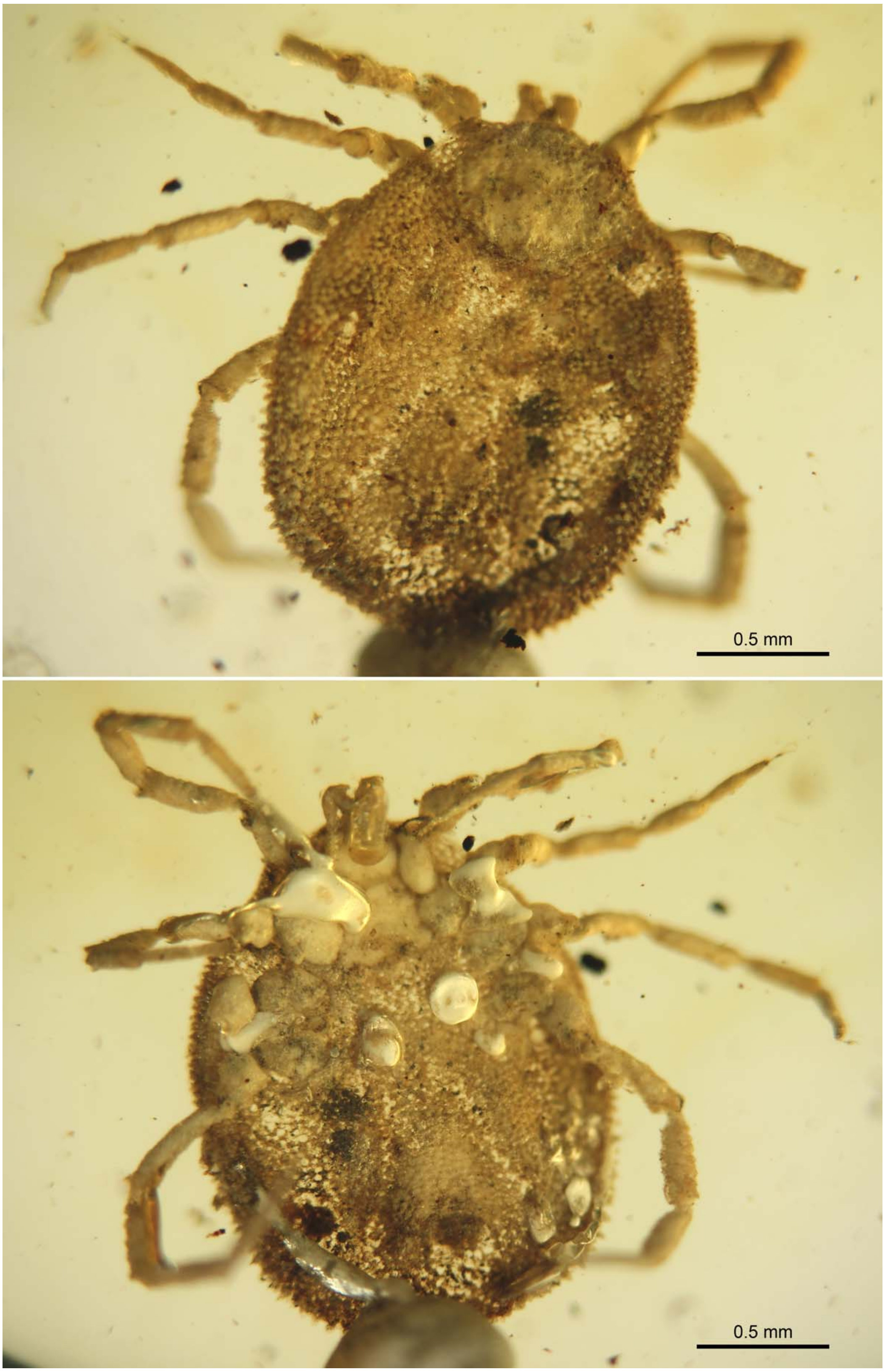

Supplementary Figure 3. Habitus of the allotype female (CM 63,007) in dorsal and ventral views. Note the integument in the dorsum showing mound-like elevations between the pits, except on the abbreviated pseudoscutum, and the lack of the right leg III.

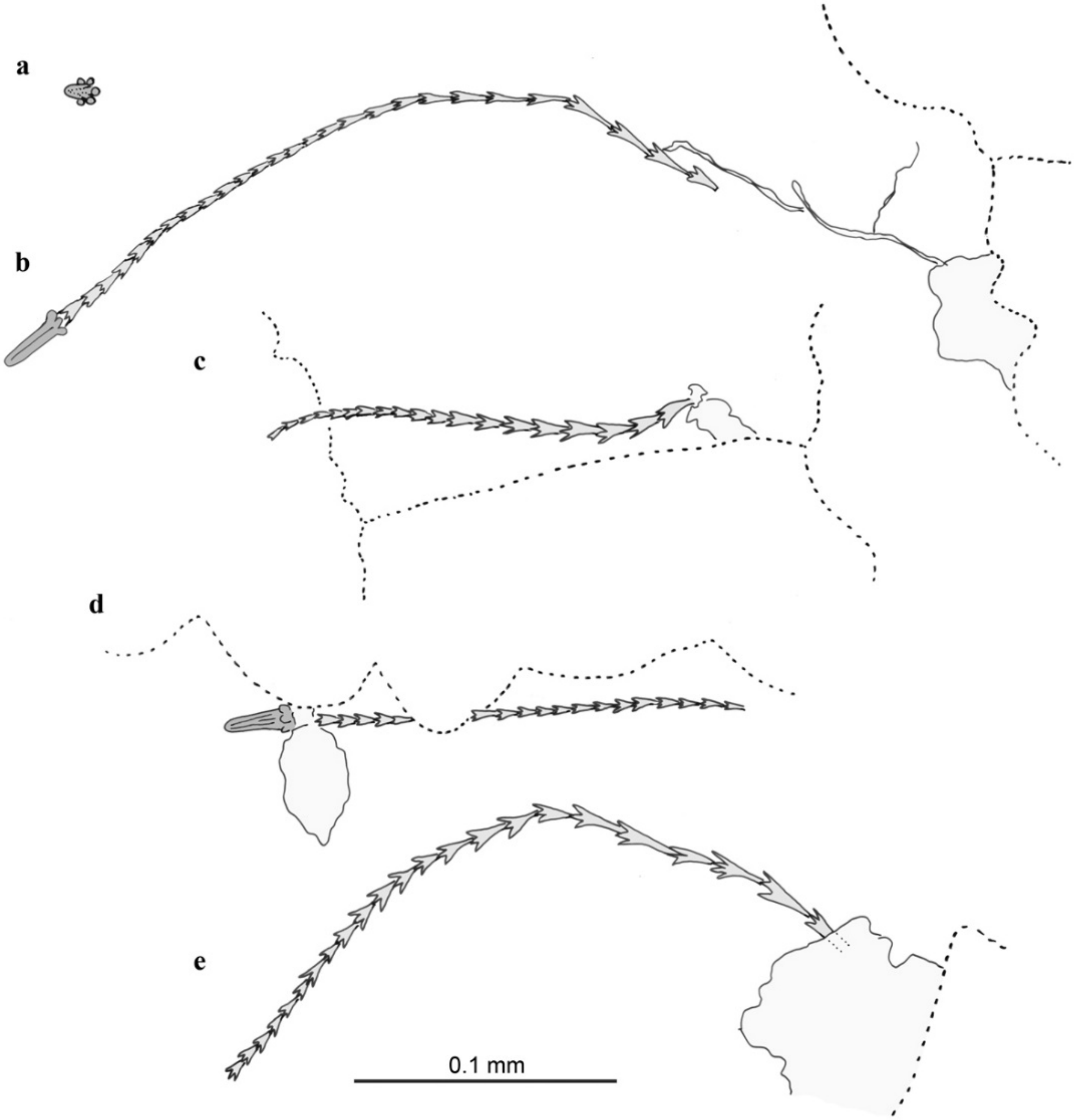

Supplementary Figure 4. Camera lucida drawings of the hastisetae present on the two deinocrotonid ticks preserved together. **a**–**b** Hastisetae preserved with its spear-head entangled in a leg of the paratype male (AMNH Bu-SA5b) (figure **a** only shows the spear-head in a frontolateral view). **c**–**e** Three of the five hastisetal remains on the holotype (AMNH Bu-SA5a), being **c** entangled in a leg, **d** in contact with the lateral body margin and preserving its spear-head, and **e** present in some debris attached to the posterior body margin (note the long basal segments indicating that it corresponds to a basal hastisetal portion). Dotted lines indicate tick body margins or legs. All to the same scale.

Supplementary Table 1- Adult external morphology of the idiosoma (except the legs) from the four tick families.

	Deinocrotonidae*	Nuttalliellidae	Ixodidae	Argasidae
**Body outline**	subcircular	subcircular	subcircular to suboval, sometimes with a straight posterior margin	subcircular to suboval, sometimes with a subtriangular and pointed anterior end
**Dorsal body integument**	with closely spaced, deep pits and mound-like elevations between pits; not extremely convoluted	closely spaced, deep pits surrounded by elevated rosettes, extremely convoluted	smooth or striate	leathery, granular and/or mammillate
**Integument setation**	absent or virtually absent; no setae associated with the pits	only present ventrally[1]	present dorsally and ventrally[2]	present dorsally and ventrally, but not always
**Scutal area**	pseudoscutum, reaching the anterior body margin in males	pseudoscutum, not reaching the anterior body margin in males[3]	scutum	scutum and pseudoscutum absent (genus *Nothoaspis* has a "false shield" termed nothoaspis)
**Scutum or pseudoscutum integument**	with closely spaced, deep pits but without mound-like elevations between pits (surface even)	mesh-like (net of deep compartments)	often punctuated to pitted	*not applicable*
**Foveal pores**	absent	absent	present	absent
**Eyes**	absent	absent	sometimes present (dorsally, on sides of scutum)	sometimes present (laterally, on supra-coxal folds)
**Genital aperture shape**	transverse	transverse	not transverse, radial	transverse
**Genital aperture distance to the capitulum**	close (in males slightly closer than in females)	close	usually more distinctly separated	close
**Genital groove**	distinct and extending posteriorly, divided medially (immediately after coxae IV)	abbreviated or absent	distinct, extending posteriorly	absent (instead, preanal groove present posteriorly)
**Anteroventral depressed area**	present, post-genital	absent	generally absent, but a pre-genital (depressed) plate present in some *Ixodes*[4]	sometimes present, post-genital[5]
**Anus position**	terminal	terminal	subterminal to terminal	mid-ventral to subterminal
**Preanal groove**	present	present	generally absent, present in *Ixodes*	generally present
**Preanal groove shape**	prolonged posteriorly, sides closing (guitar pick-shaped anal plate)	not prolonged posteriorly	if present (*Ixodes*), prolonged posteriorly, sides closing (loop-shaped anal plate) or not (inverted U-shaped anal plate)	not prolonged posteriorly or, if so, sides running to lateral body margins
**Spiracle position**	very close to the body margin, at the level of coxae IV	posterolateral to coxae IV[6]	well behind coxae IV[2,7]	between coxae III and IV[2,7]
**Spiracle size**	medium	very small[3]	generally large	medium
**Spiracular plate**	smooth	cribose[6]	smooth	smooth
**Festoons**	absent	absent	generally present	absent

* = extinct.

Supplementary Table 2- Adult external morphology of the capitulum and legs from the four tick families.

		Deinocrotonidae*	Nuttalliellidae	Ixodidae	Argasidae
CAPITULUM	**Basis capituli bordered by coxae I**	absent (anterior to coxae I)	present, entirely	present, partially and sometimes entirely	present (partially) or absent
	**Hypostome position**	subterminal (sensu Mans *et al.*)[8]	subterminal	terminal	ventral
	**Palpi length/basis capituli length**	palpi longer than basis capituli	palpi shorter than basis capituli	palpi generally longer than basis capituli	palpi subequal or longer than basis capituli
	**Palpi general shape**	gracile	stout	moderately gracile to stout[5]	gracile
	**Palpomere II**	expanded basally, distally expanded, bent distally in ventral direction	massive (not tapering basally), not bent distally	more or less tapering basally, distally expanded (top inner margin), not bent distally[5]	not tapering basally nor expanded distally, not bent distally[5]
	**Palpomere III**	tubular, tapering basally	triangular (expanded basally), broadly articulating with palpomere II	massive, broadly articulating with palpomere II	tubular
	**Palpomere IV**	terminal	terminal	subterminal, recessed in a cavity of palpomere III	terminal
	**Basis capituli surface**	smooth	irregularly transversely striated[1]	generally smooth, sometimes rugose[2,5]	striated or smooth[2,5]
	**Porose areas in basis capituli**	absent	absent	present	absent
	**Auriculae**	absent	absent[1,3]	typically found in *Ixodes*	absent
	**Cornua**	absent	present	present	absent
LEGS	**Coxal spurs**	present on all coxae	present on coxae I and II[7]	generally present on all coxae	absent
	**Ball and socket joints between trochanters and femora**	absent, but paired, ventrodistal notch-like processes present in leg articles	present	absent	absent
	**Leg ruffles**	present	present [not described, but depicted in Bedford[7] and clearly visible in Fig. 2C from Mans *et al.*[9]]	sometimes present[5]	absent
	**Haller's organ**	proximal capsule completely open, bearing several sensilla	proximal capsule with a transverse slit as an opening of its roof[1]	proximal capsule completely open (*Ixodes*) or with a transverse slit as an opening of its roof[2]	proximal capsule with a transverse slit as an opening of its roof[2]
	**Pulvilli**	poorly-developed	poorly-developed[7]	well-developed	lacking or poorly-developed[2]

* = extinct.

Supplementary Table 3- Adult ecology of the four tick families.

	**Deinocrotonidae***	**Nuttalliellidae**	**Ixodidae**	**Argasidae**
**Life style**	most likely nidicolous (see text)	nidicolous[2]	mostly non-nidicolous, some nidicolous (i.e., *Ixodes*)[2]	almost always nidicolous[2]
**Hosts**	at least feathered dinosaurs (the adult stages) (see text)	mammals, birds and reptiles[10]	mammals, birds, reptiles and amphibians (all stages)[2,11], including feathered dinosaurs in the past (at least the nymphs; the present study)	mammals, birds, reptiles and amphibians[2,11]
**Body volume increase in a feeding cycle**	around 8.5 times	5–14 times[9]	over 100 times[4]	2–10 times[4,9]
**Feeding strategy (adults)**	most likely fast feeding based on body integument and engorgement features	fast feeding, slow digestion of blood-meal[12]	prolonged feeding, rapid digestion of blood-meal[2,12]	fast feeding, generally slow digestion of blood-meal[2,12]

* = extinct.

Supplementary Note 1: Syninclusions associated to the ticks

The amber piece containing the ixodid *Cornupalpatum burmanicum* (Bu JZC-F18) also includes: remains of some feathers (including a semicomplete pennaceous feather), three bristletails (Archaeognatha), six mites (one within Mesostigmata, and two in the order Trombidiformes, including one within the Erythraeidae), one springtail of the suborder Arthropleona, one hemipteran Auchenorrhyncha, one dipteran of the family Psychodidae and another one within the Cecidomyiidae, one beetle of the family Leiodidae, coprolites, abundant plant trichomes and organic and inorganic soil particles. This fossil assemblage indicates that a resin flow reached the litter and embedded a portion of the arthropod association that inhabited the organic matter accumulated around the resiniferous trees and a few flying individuals together with soil particles; amber with these characteristics is often referred as “litter amber”[13].

The amber piece containing the deinocrotonid holotype (male) and paratype male (AMNH Bu-SA5a and AMNH Bu-SA5b, respectively) also contains dermestid hastisetae and three Heterostigmata mites, most likely within the superfamily Pyemotoidea, as syninclusions. Syninclusions associated with the engorged paratype female (CM 63,001) are: a hooded tickspider (Ricinulei) (CM 63,002), a mite of the uncommon family Smarididae (CM 63,003), two hemipterans (CM 63,004–63,005), one psocopteran (CM 63,006), legs of an arthropod, abundant plant trichomes, diverse plant debris and fungal mycelia. Syninclusions associated with the allotype (female) (CM 63,007) are: a larval mite (CM 63,008), small plant fragments with abundant trichomes (CM 63,009), isolated trichomes and inorganic soil particles.

Supplementary Note 2: Deinocrotonid body measurements

Measurements of the unengorged specimens: Body ca. 3.9 mm long from posterior margin to apex of hypostome (3.5 mm excluding hypostome), 2.7 mm greatest width and 0.5 mm greatest height. Hypostome ca. 0.35 mm long (measured in the paratype male). Palpus 0.7 mm long (estimated). Male pseudoscutum ca. 3.0 mm long, 1.8 mm greatest width (measured in the holotype); female pseudoscutum 0.8 mm long, 1.4 mm width (measured in the allotype). Legs ca. 3.5 mm long: coxa ca. 0.5 mm long, trochanter ca. 0.4 mm long, femur ca. 0.5 mm long, genu ca. 0.6 mm long, tibia ca. 0.6 mm long and tarsus ca. 0.7–0.8 mm long.

Measurements of the engorged female specimen: Body 5.9 mm long excluding hypostome, 3.7 mm greatest width and 1.9 mm greatest height. Pseudoscutum 0.5 mm long, 1.1 mm width.

Supplementary Note 3: Phylogenetic comments on Parasitiformes

The age of the order Parasitiformes is controversial. There is a wide consensus that mites (Acari) do not represent a monophyletic group[14]. On the one hand, Acariformes seem to be close to Solifugae (comprising the clade Poecilophysidea), which have a solid Palaeozoic fossil record. On the other hand, the sister group of Parasitiformes has not been clearly established, and several groups have been proposed: Pseudoscorpiones, Opiliones, Palpigradi, Poecilophysidea and Tetrapulmonata. Classically, the order Ricinulei has been proposed as the sister group of Parasitiformes (clade Cryptognomae), but the antiquity of Ricinulei is also unclear as Mesozoic fossils (from Cretaceous Burmese amber), included in the suborder Primoricinulei, are closely related to extant ricinulids (suborder Neoricinulei); meanwhile this relationship is not so clear with alleged Carboniferous ricinulids (suborder Palaeoricinulei), putatively related to the fossil order Trigonotarbida[15]. The age of the order Parasitiformes has even less consensus when using molecular clocks, because these provide a wide range of ages, from Cambrian (for the clade Parasitiformes + Tetrapulmonata)[16] to Carboniferous–Permian[17] (but see Dunlop and Selden[18]). A hypothetical age for the order Parasitiformes is not proposed herein, but we assume it possibly diversified close to the Jurassic/Cretaceous boundary as it is the most consistent and conservative approach based on direct evidence from the fossil record (Fig. 10), rather than modelling.

Current evidence suggests that Mesostigmata is sister to the rest of Parasitiformes, with Opilioacarida being sister to the clade (Holothyrida + Ixodida)[19,20]. The presence of a scutum in ticks could be considered as derived from the dorsal shield present in other parasitiform mites[12]. Under that scenario, the presence of a pseudoscutum could be considered: (1) a reduction of a fully developed scutum present in the common ancestor of all ticks (apomorphic) (proposed by Latif *et al.*[3]), the reduction of the scutum in Argasidae therefore being a convergence, or (2) the pseudoscutum is the primitive state leading to a fully developed scutum in Ixodidae (plesiomorphic). The presence of a sclerotised scutum in all life stages of the Ixodidae and the presence in nuttalliellid larvae of a sclerotised scutum similar to that observed in larval ixodids[3] support the first hypothesis.

Holothyrida larvae have a leathery integument, while adults have a heavily sclerotised integument[20]. If the presence of a scutum is a symplesiomorphy of the clade (Holothyrida + Ixodida), then the presence of a pseudoscutum could be considered a synapomorphy in Nuttalliellidae and Deinocrotonidae, while the leathery integument in adult Argasidae could be related to the retention of larval features in the adult (neoteny). The same occurs with the adaptations related to cuticle expansion that allow the increase of body volume.

Older fossil records are needed in order to clarify the phylogeny of ticks. Due to taphonomy, fossil ticks are unknown from compression deposits, which are also not good candidates for suitable

preservation of fine anatomical details. Pre-Mesozoic or pre-Cretaceous outcrops of rapid 3D mineralization preserving arthropods in fine detail, such as chert deposits, also appear unsuitable for the preservation of ticks due to their parasitic life style. The oldest amber with bioinclusions, Triassic in age, has only yielded small amber droplets with organismal inclusions in exceptional occasions[21]. The best candidate to provide older ticks is the very rich Lebanese amber, more than 25 Ma older than Burmese amber and ca. 20 Ma older than Spanish amber[22], but, interestingly, this parasitiform group has been not reported from this amber. Potential ticks in Lebanese amber should clarify the phylogeny of the clade, and could provide new clues about the age of tick appearance and radiation.

Supplementary Note 4: Feather evidence in non-avialan dinosaurs

There is consensus that feathers *sensu stricto* evolved and diversified in, at least, theropod dinosaurs before the appearance of birds or the origin of flight[23]. According to Prum's evolutionary-developmental model of the feather, with stages from I to V[24], extinct organisms exhibiting a particular stage potentially presented the previous ones. Within Theropoda, the earliest direct evidence of monofilamentous integumentary structures (FIS) is present at the basal grade of Tetanurae (Fig. 10), i.e., in phylogenetic order[25], within Megalosauroidea[26], Tyrannosauroidea[27–29], Compsognathidae[30], Ornithomimosauria[31,32], Alvarezsauridae[33] and Therizinosauridae[34,35]. Most of these FIS can be assigned to Prum's stages I (monofilamentous cylinder, often referred to as "protofeathers"), II (unbranched barbs attached by their base to a calamus) and IIIa (unbranched barbs fused along a central rachis, thus forming open vanes). Although markings in *Ornithomimus*' forearm were interpreted as insertion areas for pennaceous feathers similar to quill knobs[31], which at least would imply the presence of Prum's feather stage IV (i.e., pennaceous feathers with closed vanes due to the putative presence of interlocking barbules), this interpretation was subsequently questioned[36]. The first direct palaeontological evidence for stage IV feathers comes from basal pennaraptorans, in Oviraptorosauria[37,38]. Among paravian pennaraptorans, a wide diversity of FIS ranging from filamentous and plumulaceous to pennaceous with closed vanes (therefore from Prum's stages I to IV) is present within individuals of Scansoriopterygidae[39,40], Troodontidae[41] and Dromaeosauridae[42–44]. Especially remarkable are the dromaeosaurid *Microraptor* and the troodontid *Jianianhualong*, showing pennaceous feathers with closed asymmetrical vanes (Prum's stage V)[45, 46]. On the other hand, it is important to note that FIS are also known in some ornithischian dinosaurs[47–49]; hollowness of some of these integumentary structures suggests that they could correspond to Prum's stage I. Moreover, FIS are also known in pterosaurs, although these pycnofibres are not considered homologous to protofeathers[50].

Supplementary Note 5: Dermestid beetles and their fossil record

Dermestidae are a beetle family of more than 1,000 extant species in 50 genera[51]. Although dermestids have a cosmopolitan distribution, they are most abundant in arid or semi-arid regions. In general, Dermestidae are scavengers on a wide variety of animal and some plant remains, with remarkable ecological variation, from feeding on insect carcasses to feeding on pollen or honey in the nests of bees and wasps[51,52]. Dermestidae can be usually found in bird nests feeding on old feathers and other organic debris, or in mammal nests feeding on hair[51]. Megatomine dermestids, with hastisetae similar to those entangled on the two *Deinocroton* ticks, are especially common in bird nests, and they have been found in nests of *Passer domesticus*, *P. montanus*, *Acrocephalus palustris*, *Riparia riparia* and *Falco tinnunculus*[53–56], represented by the genera *Anthrenus*, *Globicornis*, *Trogoderma*, *Megatoma*, *Perimegatoma* and *Anthrenocerus* in these instances.

The oldest unambiguous Mesozoic record of Dermestidae is constituted by five specimens from Lebanese amber classified in a new tribe, Trinodinae, including the genus *Cretanodes* Kirejtshuk and Azar, 2009[57,58]. Nine adult specimens from younger Spanish Cretaceous amber are known[59], but none of them have been described so far. A few specimens have been found in Burmese amber[60,61], one of them an immature having multi-segmented setae[62], as well as hastisetae both attached to a parasitic wasp and isolated in the same piece of amber[63]. Recently, two new fossils in the subfamily Attageninae from New Jersey and Burmese ambers have been described[64,65].

Fossil species of Megatominae are common in Cainozoic ambers[66]. Their records are especially abundant in Baltic amber[66,67], including the larva *Trogoderma larvalis*, with preserved hastisetae[68], and in Dominican amber[66,69]. Fossils of Trinodinae: Trinodini have been scarcely found in Baltic[67] and Dominican ambers[69], and these records only include two larvae of *Apsectus* sp.

Supplementary Note 6: Additional data for Figure 10.

Phylogenetic relationships of parasitiform Acari (top of the figure) are based on Klompen[20] and Mans *et al.* [19]. Parasitiform records shown are compiled in Dunlop *et al.*[70]. For discussion on the inferred time range used for Parasitiformes, see Supplementary Note 3 above. Feeding ecology of Parasitiformes is after Walter & Proctor[71]. Phylogenetic relationships of tetanuran Dinosauria (bottom of the figure) are based on Brusatte *et al.*[25]. Inferred time ranges (ghost lineages, i.e., imposed by phylogenetic relationships) for theropod clades have been extracted from Xu *et al.*[27] and Brusatte *et al.*[72]. The inferred age for Neornithes based on targeted next-generation DNA sequencing is about 73 Ma for this clade[73]. Because the simplified tree topology does not depict this, it has been marked with an asterisk. Oldest occurrences of dinosaur groups depicted: (a) Non-pennaraptoran

tetanurans – ca. 199–190 Ma, Early Jurassic of China ("*Dilophosaurus*" *sinensis* Hu, 1993) and Antarctica (*Cryolophosaurus ellioti* Hammer & Hickerson, 1994)[74]; (b) Non-avialan pennaraptorans – ca. 161 Ma, Late Jurassic of China (e.g., *Anchiornis huxleyi* Xu *et al.*, 2009, *Aurornis xui* Godefroit *et al.*, 2013)[75]. Although *Anchiornis* was originally described as an avialan[76], subsequent works examining more conspecific material regard it as a troodontid[77,78]; a similar situation occurs for *Aurornis*[79], subsequently considered a troodontid by other authors[25]; (c) Non-ornithothoracine avialans – 150 Ma: Late Jurassic of Germany (*Archaeopteryx* von Meyer, 1861)[36,80]; (d) Enantiornithes – ca. 130 Ma: Early Cretaceous of Spain (e.g., *Noguerornis gonzalezi* Lacasa-Ruiz, 1989) and China (e.g., *Protopteryx fengningensis* Zhang & Zhou, 2000)[81,82]; (e) Non-neornithine ornithuromorphs – 131 Ma: Early Cretaceous of China (*Archaeornithura meemannae* Wang *et al.*, 2015)[83]; and (f) Neornithes (modern birds) – ca. 66–68 Ma, Late Cretaceous of Antarctica (*Vegavis iaai* Clarke *et al.*, 2005)[84].

Supplementary References

1. Keirans, J. E., Clifford, C. M., Hoogstraal, H. & Easton, E. R. Discovery of *Nuttalliella namaqua* Bedford (Acarina: Ixodoidea: Nuttalliellidae) in Tanzania and redescription of the female based on scanning electron microcopy. *Ann. Entomol. Soc. Am.* **69**, 926–932 (1976).

2. Sonenshine, D. E. 1991. *Biology of Ticks* Vols I–II (Oxford Univ. Press, New York, 1991).

3. Latif, A. A., Putterill, J. F., de Klerk, G., Pienaar, R. & Mans, B. J. *Nuttalliella namaqua* (Ixodoidea: Nuttalliellidae): First description of the male, immature stages and re-description of the female. *PLoS ONE* **7** (7), e41651 (2012).

4. Balashov, Y. S. Bloodsucking ticks (Ixodoidea)–vectors of diseases of man and animals. *Misc. Publ.*, *Entomol. Soc. Amer.* **8**, 161–376 (1972).

5. Furman, D. P. & Loomis, E. C. The Ticks of California (Acari: Ixodida). *Bull. Calif. Insect Survey*, **25**, 1–239 (1984).

6. Roshdy, M. A., Hoogstraal, H., Banaja, A. A. & El Shoura, S. M. *Nuttalliella namaqua* (Ixodoidea: Nuttalliellidae): Spiracle Structure and Surface Morphology. *Z. Parasitenkd.* **69**, 817–821 (1983).

7. Bedford, G. A. H. *Nuttalliella namaqua*, a new genus and species of tick. *Parasitol.* **23**, 230–232 (1931).

8. Mans, B. J. *et al.* Ancestral reconstruction of tick lineages. *Ticks Tick Borne Dis.* **7**, 509–535 (2016).

9. Mans, B. J., de Klerk, D., Pienaar, R. & Latif, A. A. *Nuttalliella namaqua*: a living fossil and closest relative to the ancestral tick lineage: implications for the evolution of blood-feeding in ticks. *PLoS ONE* **6** (8), e23675 (2011).

10. Mans, B. J., de Klerk, D. G., Pienaar, R. & Latif, A. A. The host preferences of *Nuttalliella namaqua* (Ixodoidea: Nuttalliellidae): a generalist approach to surviving multiple host-switches. *Exp. Appl. Acarol.* **62**, 233–240 (2014).

11. Klompen, J. S., Black, W. C. IV, Keirans, J. E. & Oliver, J. H. Jr. Evolution of ticks. *Annu. Rev. Entomol.* **41**, 141–161 (1996).

12. Mans, B. J., de Klerk, D., Pienaar, R., de Castro, M. H. & Latif, A. A. The mitochondrial genomes of *Nuttalliella namaqua* (Ixodoidea: Nuttalliellidae) and *Argas africolumbae* (Ixodoidae: Argasidae): Estimation of divergence dates for the major tick lineages and reconstruction of ancestral blood-feeding characters. *PLoS ONE* **7** (11), e49461 (2012).

13. Perrichot, V. Early Cretaceous amber from south-western France: insight into the Mesozoic litter fauna. *Geol. Acta* **2**, 9–22 (2004).

14. Pepato, A. R. & Klimov, P. B. Origin and higher-level diversification of acariform mites–evidence from nuclear ribosomal genes, extensive taxon sampling, and secondary structure alignment. *BMC Evol. Biol.* **15**, 178 (2015).

15. Selden, P. A. Revision of the fossil ricinuleids. *Trans. R. Soc. Edinburgh: Earth Sci.* **83**, 595–634 (1992).

16. Rota-Stabelli, O., Daley, A. C. & Pisani, D. Molecular timetrees reveal a Cambrian colonization of land and a new scenario for ecdysozoan evolution. *Curr. Biol.* **23**, 392–398 (2013).

17. Jeyaprakash, A. & Hoy, M. A. First divergence time estimate of spiders, scorpions, mites and ticks (subphylum: Chelicerata) inferred from mitochondrial phylogeny. *Exp. Appl. Acarol.* **47**, 1–18 (2009).

18. Dunlop, J. A. & Selden, P. A. Calibrating the chelicerate clock: a paleontological reply to Jeyaprakash and Hoy. *Exp. App. Acarol.* **48**, 183–197 (2009).

19. Mans, B. J., de Klerk, D. G., Pienaar, R., de Castro, M. H. & Latif, A. A. Next-generation sequencing as means to retrieve tick systematic markers, with the focus on *Nuttalliella namaqua* (Ixodoidea: Nuttalliellidae). *Ticks Tick Borne Dis.* **6**, 450–462 (2015).

20. Klompen, H. Holothyrids and ticks: new insights from larval morphology and DNA sequencing, with the description of a new species of *Diplothyrus* (Parasitiformes: Neothyridae). *Acarologia* **50**, 269–285 (2010).

21. Schmidt, A. R. *et al*. Arthropods in amber from the Triassic Period. *PNAS* **109**, 14796–14801 (2012).

22. Maksoud, S. *et al*. Revision of "Falaise de BLANCHE" (Lower Cretaceous) in Lebanon, with the definition of a Jezzinian Regional Stage. *Carnets Géol.* **14**, 401–427 (2014).

23. Prum, R. O. & Brush, A. H. Which came first, the feather or the bird? *Sci. Am.* **288**, 60–69 (2014).

24. Prum, R. O. Development and evolutionary origin of feathers. *J. Exp. Zool.* **285**, 291–306 (1999).

25. Brusatte, S. L., Lloyd, G. T., Wang, S. C. & Norell, M. A. Gradual assembly of avian body plan culminated in rapid rates of evolution across the dinosaur-bird transition. *Curr. Biol.* **24**, 2386–2392 (2014).

26. Rauhut, O. W., Foth, C., Tischlinger, H. & Norell, M. A. Exceptionally preserved juvenile megalosauroid theropod dinosaur with filamentous integument from the Late Jurassic of Germany. *PNAS* **109**, 11746–11751 (2012).

27. Xu, X. *et al*. An integrative approach to understanding bird origins. *Science* **346**, 1253293 (2014).

28. Xu, X. *et al*. Basal tyrannosauroids from China and evidence for protofeathers in tyrannosauroids. *Nature* **431**, 680–684 (2004).

29. Xu, X. *et al*. A gigantic feathered dinosaur from the Lower Cretaceous of China. *Nature* **484**, 92–95 (2012).

30. Currie, P. J. & Chen, P. J. Anatomy of *Sinosauropteryx prima* from Liaoning, northeastern China. *Can. J. Earth Sci.* **38**, 1705–1727 (2001).

31. Zelenitsky, D. K. *et al*. Feathered non-avian dinosaurs from North America provide insight into wing origins. *Science* **338**, 510–514 (2012).

32. van der Reest, A. J., Wolfe, A. P. & Currie, P. J. A densely feathered ornithomimid (Dinosauria: Theropoda) from the Upper Cretaceous Dinosaur Park Formation, Alberta, Canada. *Cret. Res.* **58**, 108–117 (2016).

33. Schweitzer, M. H. *et al*. Beta-Keratin Specific Immunological Reactivity in Feather-like Structures of the Cretaceous Alvarezsaurid, *Shuvuuia deserti. J. Exp. Zool. B (Mol. Del. Evol.)* **285**, 146–157 (1999).

34. Xu, X., Tang, Z. L. & Wang, X. L. A therizinosauroid dinosaur with integumentary structures from China. *Nature* **399**, 350–354 (1999).

35. Xu, X., Zheng, X. & You, H. A new feather type in a nonavian theropod and the early evolution of feathers. *PNAS* **106**, 832–834 (2009).

36. Foth, C., Tischlinger, H. & Rauhut, O. W. New specimen of *Archaeopteryx* provides insights into the evolution of pennaceous feathers. *Nature* **511**, 79–82 (2014).

37. Qiang, J., Currie, P. J., Norell, M. A. & Shu-An, J. Two feathered dinosaurs from northeastern China. *Nature* **393**, 753–761 (1998).

38. Xu, X., Zheng, X. & You, H. Exceptional dinosaur fossils show ontogenetic development of early feathers. *Nature* **464**, 1338–1341 (2010).

39. Zhang, F., Zhou, Z., Xu, X., Wang, X. & Sullivan, C. A bizarre Jurassic maniraptoran from China with elongate ribbon-like feathers. *Nature* **455**, 1105–1108 (2008).

40. Xu, X. *et al*. A bizarre Jurassic maniraptoran theropod with preserved evidence of membranous wings. *Nature* **521**, 70–73 (2015).

41. Godefroit, P. *et al*. Reduced plumage and flight ability of a new Jurassic paravian theropod from China. *Nat. Commun*. **4**, 1394 (2013).

42. Xu, X., Wang, X. L. & Wu, X. C. A dromaeosaurid dinosaur with a filamentous integument from the Yixian Formation of China. *Nature* **401**, 262–266 (1999).

43. Xu, X., Zhou, Z. H. & Prum, R. O. Branched integumental structures in *Sinornithosaurus* and the origin of feathers. *Nature* **410**, 200–204 (2001).

44. Lü, J. & Brusatte, S. L. A large, short-armed, winged dromaeosaurid (Dinosauria: Theropoda) from the Early Cretaceous of China and its implications for feather evolution. *Sci. Rep.* **5**, 11775 (2015).

45. Xu, X. *et al*. Four-winged dinosaurs from China. *Nature* **421**, 335–340 (2003).

46. Xu, X. *et al.* Mosaic evolution in an asymmetrically feathered troodontid dinosaur with transitional features. *Nat. Commun.* **8**, 14972 (2017).

47. Mayr, G. Peters, S. D., Plodowski, G. & Vogel, O. Bristle-like integumentary structures at the tail of the horned dinosaur *Psittacosaurus. Naturwissenschaften* **89**, 361–365 (2002).

48. Zheng, X. T., You, H. L., Xu, X. & Dong, Z. M. An Early Cretaceous heterodontosaurid dinosaur with filamentous integumentary structures. *Nature* **458**, 333–336 (2009).

49. Godefroit, P. *et al*. A Jurassic ornithischian dinosaur from Siberia with both feathers and scales. *Science* **345**, 451–455 (2014).

50. Kellner, A. W. *et al*. The soft tissue of *Jeholopterus* (Pterosauria, Anurognathidae, Batrachognathinae) and the structure of the pterosaur wing membrane. *Proc. Roy. Soc. Lond. B Biol. Sci.* **277**, 321–329 (2010).

51. Lawrence, J. F. & Ślipiński, S. A. in *Morphology and Systematics (Elateroidea, Bostrichiformia, Cucujiformia partim)* Vol. 2 (eds Leschen, R. A. B. & Beutel, R. G.) 198–206 (de Gruyter, Berlin, 2010).

52. Kingsolver, J. M. in *American Beetles* Vol. 2 (eds Arnett R. H. Jr., Thomas, M. C., Skelley, P. E. & Frank, J. H.) 228–232 (CRC Press, 2002).

53. Šustek, Z. & Krištofik, J. Beetles (Coleoptera) in nests of house and tree sparrows (*Passer domesticus* and *P. montanus*). *Biologia, Bratislava* **58**, 953–965 (2003).

54. Krištofik, J., Šustek, Z. & Gadjoš, P. Arthropods in nests of the Sand Martin (*Riparia riparia* Linnaeus, 1758) in South Slovakia. *Biologia, Bratislava* **49**, 683–690 (1994).

55. Krištofik, J., Mašán, P. & Šustek, Z. Arthropods in the nests of marsh warblers (*Acrocephalus palustris). Biologia, Bratislava* **60**, 171–177 (2005).

56. Kaľavský, M., Fenďa, P. & Holecová, M. Arthropods in the nests of Common Kestrel (*Falco tinnunculus*). *Slovak. Rapt. J.* **3**, 29–33 (2009).

57. Kirejtshuk, A. G., Azar, D., Tafforeau, P., Boistel, R. & Fernandez, V. New beetles of Polyphaga (Coleoptera, Polyphaga) from Lower Cretaceous Lebanese amber. *Denisia* **26**, 119–130 (2009).

58. Kirejtshuk, A. G. & Azar, D. Current knowledge of Coleoptera (Insecta) from the Lower Cretaceous Lebanese amber and taxonomical notes for some Mesozoic groups. *Terr. Arthropod Rev.* **6**, 103–134 (2013).

59. Peris, D., Ruzzier, E., Perrichot, V. & Delclòs, X. Evolutionary and paleobiological implications of Coleoptera (Insecta) from Tethyan-influenced Cretaceous ambers. *Geosci. Front.* **7**, 695–706 (2016).

60. Grimaldi, D. & Engel, M. S. *Evolution of the Insects* (Cambridge University Press, New York, 2005).

61. Grimaldi, D. A., Engel, M. S. & Nascimbene, P. C. Fossiliferous Cretaceous amber from Myanmar (Burma): its rediscovery, biotic diversity, and paleontological significance. *Am. Mus. Novit.* **3361**, 1–71 (2002).

62. Cockerell, T. D. A. Arthropods in Burmese amber. *Psyche* **24**, 40–45 (1917).

63. Poinar, G. & Poinar, R. Ancient hastisetae of Cretaceous carrion beetles (Coleoptera: Dermestidae) in Myanmar amber. *Arthropod Struct. Dev.* **45** (6), 642–645 (2016).

64. Peris, D. & Háva, J. New species from Late Cretaceous New Jersey amber and stasis in subfamily Attageninae (Insecta: Coleoptera: Dermestidae). *J. Paleontol.* **90**, 491–498 (2016).

65. Cai, C., Háva, J. & Huang, D. The earliest *Attagenus* species (Coleoptera: Dermestidae: Attageninae) from Upper Cretaceous Burmese amber. *Cret. Res.* **72**, 95–99 (2017).

66. Háva, J. Dermestidae World (Coleoptera). www.dermestidae.wz.cz/main.html (2014).

67. Háva, J. & Damgaard, A. L. A New Species of *Globicornis* (*Hadrotoma*) (Coleoptera, Dermestidae, Megatominae) from Baltic Amber. *Vest. Zool.* **49**, 373–376 (2015).

68. Háva, J., Prokop, J. & Herrmann, A. New fossil dermestid beetles (Coleoptera: Dermestidae) from the Baltic amber. *Acta Soc. Zool. Bohem.* **69**, 281–287 (2006).

69. Poinar, G. O. & Háva, J. New Dermestidae (Coleoptera) from Dominican amber. *Palaeodiv.* **8**, 1–11 (2015).

70. Dunlop, J. A., Penney, D. & Jekel, D. in *World Spider Catalog* version 18.5 (Natural History Museum Bern, online at http://wsc.nmbe.ch, 2017).

71. Walter, D. E. & Proctor, H. C. *Mites: ecology, evolution and behaviour*. Sydney: UNSW Press (1999).

72. Brusatte, S. L., O'Connor, J. K. & Jarvis, E. D. The origin and diversification of birds. *Curr. Biol.* **25**, R888–R898 (2015).

73. Prum, R. O. *et al.* A comprehensive phylogeny of birds (Aves) using targeted next-generation DNA sequencing. *Nature* **526**, 569–573 (2015).

74. Carrano, M. T., Benson, R. B. & Sampson, S. D. The phylogeny of Tetanurae (Dinosauria: Theropoda). *J. Syst. Palaeontol.* **10**, 211–300 (2012).

75. Liu, Y. *et al.* Timing of the earliest known feathered dinosaurs and transitional pterosaurs older than the Jehol Biota. *Palaeogeo. Palaeoclim. Palaeoeco.* **323**, 1–12 (2012).

76. Xu, X. *et al.* A new feathered maniraptoran dinosaur fossil that fills a morphological gap in avian origin. *Chinese Sci. Bull.* **54**, 430–435 (2009).

77. Hu, D., Hou, L., Zhang, L. & Xu, X. A pre-*Archaeopteryx* troodontid theropod from China with long feathers on the metatarsus. *Nature* **461**, 640–643 (2009).

78. Li, Q. *et al.* Plumage color patterns of an extinct dinosaur. *Science* **327**, 1369–1372 (2010).

79. Godefroit, P. *et al*. A Jurassic avialan dinosaur from China resolves the early phylogenetic history of birds. *Nature* **498**, 359–362 (2013).

80. von Meyer, H. V. *Archaeopteryx lithographica* (Vogel-Feder) und *Pterodactylus* von Solnhofen. *N. Jb. Mineral.*, *Geognosie, Geol. und Petrefaktenkunde* **1861**, 678–679 (1861).

81. Chiappe, L. M. & Lacasa-Ruíz, A. in *Mesozoic Birds, Above the Heads of Dinosaurs* Chap. 10 (eds Chiappe, L. M. & Witmer, L. M.) 230–239 (Univ. California Press, 2002).

82. Zhang, F. & Zhou, Z. A primitive enantiornithine bird and the origin of feathers. *Science* **290**, 1955–1959 (2000).

83. Wang, M. *et al*. The oldest record of Ornithuromorpha from the early cretaceous of China. *Nat. Commun.* **6**, 6987 (2015).

84. Clarke, J. A., Tambussi, C. P., Noriega, J. I., Erickson, G. M. & Ketcham, R. A. Definitive fossil evidence for the extant avian radiation in the Cretaceous. *Nature* **433**, 305–308 (2005).
