# Supplementary material for: Ticks parasitised feathered dinosaurs as revealed by Cretaceous amber assemblages
Source: Nat Commun. 2017 Dec 12;8:1924. doi: 10.1038/s41467-017-01550-z (PMC5727220; doi:10.1038/s41467-017-01550-z)
Supplement: Supplementary file 3 — Description of Additional Supplementary Files [file 41467_2017_1550_MOESM3_ESM.pdf]

File Name: Supplementary Movie 1
Description: **3D anatomical model of the tick holotype.** Movie showing the 3D reconstruction of the deinocrotonid holotype rotating based on CT-scans (body length ca. 3.9 mm) (Artist: Oscar Sanisidro).
